# Social network size and endorsement of political violence in the US

**DOI:** 10.1186/s40621-024-00540-2

**Published:** 2024-10-17

**Authors:** Julia P. Schleimer, Paul M. Reeping, Sonia L. Robinson, Garen J. Wintemute

**Affiliations:** 1 Violence Prevention Research Program, Department of Emergency Medicine, University of California, Davis, Sacramento, CA USA; 2 California Firearm Violence Research Center, Sacramento, CA USA

**Keywords:** Political violence, Violence and society, Racism

## Abstract

**Background:**

In recent years, the United States (US) has witnessed a rise in political violence. Prior research has found that an individual’s social network is associated with their likelihood of engaging in various forms of violence, but research on social networks and political violence in the US context is limited. This study examined associations between social network size and endorsement of political violence in a recent nationally representative survey and explored how the relationship varied by use of social media as a major news source, perceptions of the government as an enemy, and membership in a marginalized or privileged racial or ethnic group.

**Methods:**

This was a nationally representative cross-sectional survey study of adults aged 18 and older in the US, administered from May 13-June 2, 2022. The exposure was social network size, defined by the number of strong social connections. We examined three violence-related outcomes: support for non-political violence, support for political violence, and personal willingness to engage in political violence. We estimated prevalence ratios for associations using survey-weighted Poisson regression with robust standard errors, adjusting for hypothesized confounders and including interaction terms to examine effect measure modification.

**Results:**

The sample included 8,620 respondents. Median age was 48.4 years (95% CI = 47.9–48.8), 51.5% were female (95% CI = 50.4–52.7%), and 62.7% self-identified as non-Hispanic White (95% CI = 61.4–65.9%). In adjusted models, those with zero strong connections were more likely than those with 1–4 strong social connections to consider political violence usually/always justified in general (PR = 2.43, 95% CI = 1.47–4.01). Those with 50 + strong connections were more likely than those with 1–4 strong social connections to consider political violence usually/always justified in at least one situation (PR = 1.19, 95% CI = 1.03–1.37) and were more likely to report being willing to personally use political violence (PR = 1.52, 95% CI = 1.13–2.04). Associations varied somewhat by social media use, perceptions of the government as an enemy, and racialized identity.

**Conclusions:**

Individuals who reported very few and very many strong social connections were more likely than others to support political violence or be personally willing to engage in it in one form or another. Findings point toward potential intervention and prevention opportunities.

**Supplementary Information:**

The online version contains supplementary material available at 10.1186/s40621-024-00540-2.

## Background

In recent years, the United States (US) has witnessed an increase in political violence, i.e., the use of force to advance political objectives (Armed Conflict Location & Event Data Project 2024; Kleinfeld 2021; Parker et al. 2023; Wintemute 2021). This trend, marked by an increase in extremist ideologies and politically motivated attacks, is a growing concern. Political violence challenges the fabric of democracy and the public functions democracy upholds and may, directly and indirectly, harm population health (Besley and Kudamatsu 2006; de Jong et al. 2015; Hamber 2009; Jamali et al. 2000; Kleinfeld 2022; Leatherman and Thomas 2009; Papadopoulos 2004; Pedersen 2002; Safaei 2006; Sousa 2013). Indeed, political violence may have important direct consequences for physical and mental health (e.g., injury, anxiety) and, if politically-motivated violence affects political actors and regimes in anti-democratic ways, it may have indirect consequences for political factors that shape social determinants of health (e.g., housing, employment, environmental conditions, food access, etc.) by structuring relationships, distributing resources, and administering power (Dawes 2022). It is therefore important to understand factors associated with support for and willingness to engage in political violence.

Prior research has found that an individual’s social network, including network size, is associated with their likelihood of engaging in various forms of violence (Levendosky et al. 2004; Niño et al. 2017; Papachristos et al. 2013, 2015; Pfundmair 2024; Sierra-Arévalo and Papachristos 2017; Tita and Radil 2011). Notably, both extremes of social network size—very few and very many connections—have been associated with movement toward extremism and endorsement of terrorism and violence. People who lack strong social connections have been identified as more susceptible to extremist ideologies and more likely than others to use violence (Capellan 2015; McCauley et al. 2013; Pfundmair 2024; Phillips 2012; Reid Meloy and Yakeley 2014), suggesting a potential etiologic role of social isolation in politically-motivated violence. At the same time, personal identification with antisocial groups is associated with political violence (Littman and Paluck 2015), and the law of “group polarization” (Sunstein 1999) suggests that when large groups of people with similar views unite, their views are more likely to radicalize and drift toward extremism, potentially leading to political violence (Littman and Paluck 2015; Piazza 2023; Pynchon and Borum 1999). Most prior research on social networks and political violence specifically has been conducted outside of the US (Bélanger et al. 2020; Everton 2016; Fafchamps and Vicente 2013; Perliger and Pedahzur 2011), with no recent empirical analyses, to our knowledge, of social network size and political violence in the US. Because social networks and violence are shaped in part by historical and context-dependent structural, social, and cultural factors which vary from country to country and over time, it is important to understand this association in the contemporary US context.

**Fig. 1:**
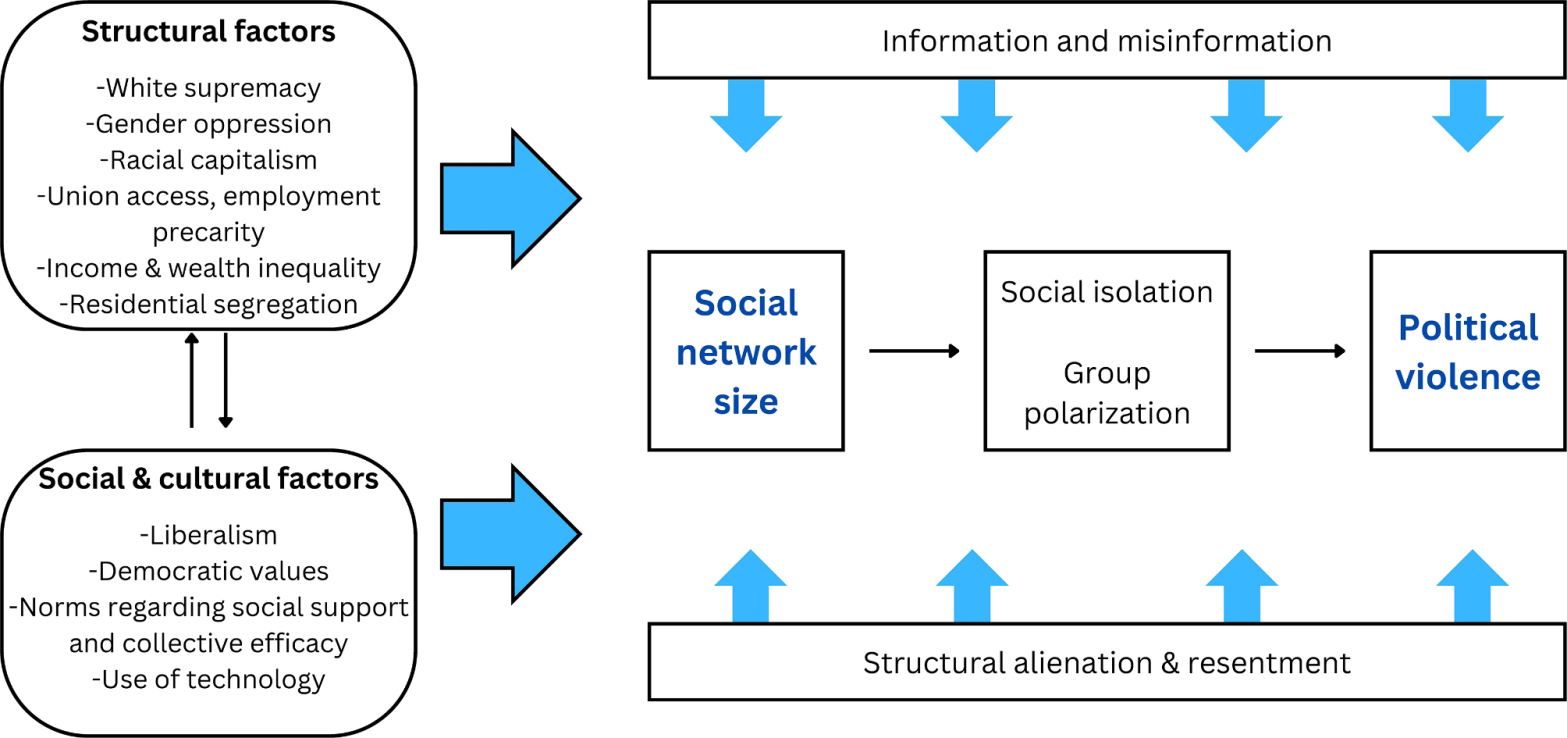
Conceptual model

We developed a conceptual model to illustrate such structural, social, and cultural factors, hypothesized mechanisms linking social network size with political violence, as well as factors that may modify this association (Fig. 1). Specifically, we examine three factors hypothesized to modify the social network-political violence association to the extent that they compound or intensify feelings of social isolation or group polarization: (1) social media use as a source of news and information, (2) perceptions of the government as an enemy, and (3) membership in a systemically marginalized or privileged racial or ethnic group. We focus on social media as a source of news and information because the digital age has amplified the possibility of online radicalization and the power of online platforms to shape individuals’ understanding of the world and their position in it (through information or misinformation) as well as their affiliations with radical groups, both online and offline (Alava et al. 2017; Awan 2017; Barrett 2024; Del Vicario et al. 2016; Pauwels and Hardyns 2018). We focus on perceptions of the government as an enemy and membership in a systemically marginalized or privileged racial or ethnic group as potential indicators of structural alienation (feelings of “powerlessness, estrangement and isolation” embedded in unequal societies (Øversveen et al. 2022)) vis-à-vis the government and one’s social position in society, respectively. When individuals perceive the government as failing to protect their interests, priorities, or social identity, they may feel increasingly resentful and inclined to endorse or enact political violence (Kleinfeld 2021). Likewise, in light of the legacy and continuation of White supremacy in the US, both dominant and oppressed racialized groups may feel disenfranchised (whether perceived or actual) in the course of conflict to uphold or dismantle socially-constructed racial and ethnic hierarchies in the US (Francis et al. 2021; Kleinfeld 2021; Metzl 2019). Feelings of racial disenfranchisement may lead to anger, resentment, and lashing out; Bonilla-Silva describes such “socially engendered emotions in racialized societies” as “racialized emotions” (Bonilla-Silva 2019).

Building on our earlier work (Wintemute et al. 2023, 2024), this study (1) examines associations between social network size and endorsement of political violence in a recent nationally representative survey, and (2) explores how this relationship varies by use of social media as a major source of news, perceptions of the government as an enemy, and self-identified membership in systemically marginalized or privileged racial or ethnic group. Results from this study will contribute to a broader understanding of political violence and inform intervention efforts.

## Methods

### Data

This was a nationally representative cross-sectional survey study of adults aged 18 and older in the US. Data were from the 2022 Life in America Survey, which was designed by the authors and administered online in English and Spanish from May 13 to June 2, 2022, by the survey research firm Ipsos (Ipsos 2023). Survey respondents were drawn from the Ipsos KnowledgePanel, an online research panel with members recruited through address-based probability sampling that has been widely used in population-based research (Kravitz-Wirtz et al. 2021; Miller et al. 2022;  Schleimer et al. 2019; Wintemute et al. 2022). Invitations were sent via e-mail with e-mail and telephone reminders beginning three days later. Recruited adults were provided a web-enabled device and free internet, if needed. Survey weights were applied to adjust for initial probability of selection and for survey-specific non-response and over- or under-coverage using design weights with post-stratification raking ratio adjustments. With weighting, the sample is designed to statistically represent the non-institutionalized adult population of the US as reflected in the 2021 March supplement of the Current Population Survey, the most recent release at the time of our survey. Participants were provided informed consent language before accessing the questionnaire that concluded, “(by) continuing, you are agreeing to participate in this study.” Additional details about the survey methods are described elsewhere (Wintemute et al. 2023). The study is reported following 2021 American Association for Public Opinion Research guidelines (AAPOR 2022), and was approved by the University of California, Davis Institutional Review Board.

### Exposure

The main exposure was social network size, defined by the number of strong social connections (“the people with whom you have a personal or work relationship and communicate with regularly”) that respondents reported in response to the question: “How many people are there, other than yourself, with whom you have a strong connection?” Response options were 0, 1–4, 5–9, 10–19, 20–49, and 50 or more.

### Outcomes

We examined three types of violence-related outcomes: (1) support for “force or violence,” hereafter “non-political violence” (with force or violence defined as “physical force strong enough that it could cause pain or injury to a person”); (2) support for political violence (defined as “force or violence to achieve political objectives”); and (3) personal willingness to engage in political violence. While the focus of our study was on political violence, we included non-political violence to assess the specificity of our findings.

Support for non-political violence was measured in 7 situations. Support for political violence was measured both in general and in 17 situations (each respondent saw 13). Personal willingness to engage in political violence was measured for 16 types and targets of political violence, three of which referred to social networks: “use force or violence as part of a group of people who share your beliefs,” “use force or violence on your own, as an individual,” and “organize a group of people who share your beliefs to use force or violence.”

Response options for non-political and political violence support were “always,” “usually,” “sometimes,” and “never” justified. We grouped responses into usually/always and never/sometimes. Response options for personal willingness to engage in political violence were “completely,” “very,” “somewhat,” and “not at all” willing. We grouped responses into very/completely and not at all/somewhat willing. Respondents were categorized as supportive of or willing to use violence if they indicated usually/always justified or very/completely willing, respectively, for at least one situation, type, or target presented.

### Modifiers

We examined effect measure modification of the relationship between social network size and violence outcomes by whether respondents reported any social media platform as a major source of news, whether they reported viewing at least one government institutions as an enemy, and whether they self-identified membership in a systemically marginalized or privileged racial or ethnic group.

Social media as a source of news was measured by asking respondents: “How much do you use each of the following Internet sites and apps as a source of news and information?” for 15 social media platforms, with response options: not a source, minor source, and major source. Social media platforms included: Facebook/Meta, Twitter (now X), LinkedIn, Parler, YouTube, Instagram, Tik Tok, Reddit, Rumble, 8chan/8kum, Telegram, WhatsApp, Signal, Truth Social, and Gab. We categorized respondents by whether they indicated that at least one social media platform was a major source of news vs. none.

Perceptions of the government as an enemy were measured by asking respondents: ‘On a scale of 1 to 5—where “1” means you think the institution is your enemy and “5” means you think the institution is your friend—where on this scale would you place yourself?’ for 7 institutions. Institutions included: the federal government, state government, local government, police and sheriffs, courts and judges, military and national guard, and state and local health departments. We categorized respondents by whether they indicated at least one government institution was a 1 or 2 on the friend-enemy scale vs. none (i.e., all institutions were a 3, 4 or 5 on the friend-enemy scale).

Membership in a systemically marginalized or privileged racial or ethnic group was self-reported (check all that apply) as part of KnowledgePanel profile data. Because the US was built on and continues to be shaped by White supremacy ideology and culture (Bonilla-Silva 1997), we dichotomized racial or ethnic group identity as non-Hispanic White vs. non-White race or Hispanic ethnicity including two or more races (hereafter “non-White”) for analyses.

Survey questions and response options for all key variables are in Supplementary Material 1: Table S1.

### Analysis

We calculated unweighted counts and weighted percentages and 95% confidence intervals (CIs). We estimated prevalence ratios (PRs) and adjusted prevalence ratios (aPR) for associations using survey-weighted Poisson regression with robust standard errors. Hypothesized confounders included the following self-reported variables: individual age (measured continuously in years), sex (female and male), annual household income (less than $10,000; $10,000 to $24,999; $25,000 to $49,999; $50,000 to $74,999; $75,000 to $99,999; $100,000 to $149,999; $150,000 or more), education (no high school or General Educational Development [GED], high school or GED, some college or associates, bachelor’s degree, master’s degree or higher), employment status (working full time, working part time, not working), and political party affiliation (strong Republican, not strong Republican, leans Republican, undecided/independent/other, leans Democrat, not strong Democrat, and strong Democrat). Regression models treated social network size of 1–4 connections as the referent since this was the largest group. We tested for interactions using likelihood ratio tests with alpha of < 0.20, as suggested by Jewell 2004, since interactions reduce power to detect associations which may be of scientific interest (Jewell 2004). Analyses were conducted in Stata, release 18.0 (StataCorp LLC).

### Secondary and sensitivity analysis

In secondary analyses, we examined the associations of social network size with each of the 17 situations in which respondents might justify political violence and each of the 16 types and targets of violence for which respondents might be personally willing to engage in political violence.

We also defined social networks both by size and uniformity. We used the question: “Thinking again about the people with whom you have a strong connection, what percentage of them share your beliefs about the use of force or violence to advance important political objectives that they support?” Response options were none/almost none of them, less than half of them, about half of them, more than half of them, all/nearly all of them, and don’t know. We defined shared beliefs dichotomously, by those who reported that half or less than half of their close connections share their beliefs about political, including don’t know, and those who reported that more than half of their close connected share their beliefs about political violence. We then created a variable representing the intersection of social network size and shared beliefs.

In a sensitivity analysis, we repeated the main analysis after removing 447 individuals who indicated that any of two fake social media platforms were a major or minor source of news and information (included in the survey as attention checks). These fake social media platforms were not used to construct the variable categorizing social media as a major source of news, but we conducted this sensitivity analysis because individuals who endorsed these fake sources may have responded unreliably to other questions.

We further examined effect measure modification by perceptions of the government as an enemy using an alternative cutoff in which respondents were categorized by whether they indicated at least one government institution was a 1 on the friend-enemy scale vs. none (i.e., all institutions were a 2, 3, 4 or 5 on the friend-enemy scale).

Finally, we examined effect measure modification by continuous versions of modifiers to assess possible dose response.

## Results

### Description of study sample

Of 15,449 individuals invited to participate, 8,620 completed the survey (55.8% completion percentage). Median time to survey completion was 15.7 min (interquartile range = 11.4–23.0). Item nonresponse was less than 2%. Prior work compared survey respondents and non-respondents, findings some differences in race and ethnicity, marital status; education, income, and employment (Wintemute et al. 2023).

Median age of respondents was 48.4 years (95% CI = 47.9–48.8), 51.5% were female (95% CI = 50.4–52.7%), 75.5% (95% CI = 74.4–76.7%) identified as White (62.7% self-identified as non-Hispanic White), 16.9% identified as Hispanic (95% CI = 15.9–17.9%), and 12.9% identified as Black or African American (95% CI = 12.0-13.8%) (Table 1). A plurality of respondents reported 1–4 strong social connections (34.5%, 95% CI = 33.3–35.6%), and less than 5% of respondents reported 50+ (4.2%, 95% CI = 3.7–4.7%) or zero strong social connections (3.7%, 95% CI = 3.3–4.3%).

**Table 1:** Description of study sample, *N* = 8620

	Unweighted No.	Weighted %	Weighted 95% CI
Total	8620	100	--
**Age**			
18–29	924	20.1	(18.9–21.3)
30–44	1921	25.6	(24.6–26.7)
45–59	2027	23.9	(22.9–24.9)
60+	3748	30.4	(29.5–31.4)
**Sex**			
Female	4373	51.5	(50.4–52.7)
Male	4247	48.5	(47.3–49.6)
**Race and ethnicity** ^a^			
American Indian or Alaska Native	231	2.7	(2.3–3.2)
Asian Indian	66	1.1	(0.9–1.5)
Black or African American	936	12.9	(12.0-13.8)
Chinese	138	2.1	(1.7–2.5)
Filipino	69	0.9	(0.7–1.2)
Guamanian	4	< 0.1	(< 0.1–0.1)
Hawaiian	14	0.2	(0.0-0.4)
Hispanic	1084	16.9	(15.9–17.9)
Japanese	63	0.7	(0.5-1.0)
Korean	39	0.6	(0.4–0.8)
Other Asian	40	0.9	(0.6–1.2)
Other Pacific Islander	5	< 0.1	(< 0.1–0.2)
Samoan	8	< 0.1	(< 0.1–0.2)
Some other race	162	2.6	(2.2–3.1)
Vietnamese	18	0.3	(0.2–0.5)
White	7,015	75.5	(74.4–76.7)
**Household income**			
Less than $10,000	272	3.9	(3.4–4.4)
$10,000 to $24,999	745	9	(8.3–9.7)
$25,000 to $49,999	1469	17.0	(16.1–17.9)
$50,000 to $74,999	1414	16.3	(15.5–17.2)
$75,000 to $99,999	1214	13.2	(12.4–14.0)
$100,000 to $149,999	1500	17.9	(17.0-18.8)
$150,000 or more	2006	22.8	(21.8–23.8)
**Education**			
No high school or GED	542	9.5	(8.7–10.3)
High school or GED	2,158	28.3	(27.2–29.4)
Some college or associates	2,362	27.1	(26.0-28.1)
Bachelor’s degree	1,951	19.7	(18.8–20.6)
Master’s degree or higher	1,605	15.4	(14.7–16.2)
**Employment status**			
Working full-time	3888	48.0	(46.8–49.2)
Working part-time	1132	14.3	(13.4–15.2)
Not working	3600	37.7	(36.6–38.9)
**Political party affiliation**			
Strong Republican	1436	15.4	(14.6–16.2)
Not strong Republican	925	10.5	(9.8–11.2)
Leans Republican	1493	16.8	(15.9–17.7)
Undecided/Independent/Other	309	3.9	(3.4–4.4)
Leans Democrat	1716	21.4	(20.4–22.5)
Not strong Democrat	1095	14.1	(13.2–15.0)
Strong Democrat	1608	17.6	(16.7–18.5)
**Number of strong social connections**			
0	288	3.7	(3.3–4.3)
1–4	2830	34.5	(33.3–35.6)
5–9	2615	30.0	(28.9–31.1)
10–19	1650	18.3	(17.4–19.2)
20–49	755	7.9	(7.3–8.5)
50+	386	4.2	(3.7–4.7)
**Consider non-political violence usually/always justified in one or more situations** ^b^			
No	1017	12.6	(11.8–13.4)
Yes	7545	86.5	(85.7–87.4)
**Consider political violence usually/always justified “in general”**			
No	8403	96.6	(96.0-97.1)
Yes	189	3.0	(2.6–3.6)
**Consider political violence usually/always justified in one or more situations** ^b^			
No	5850	67.2	(66.1–68.3)
Yes	2770	32.8	(31.7–33.9)
**Very/completely willing to personally use political violence for one or more type or target of violence** ^b^			
No	7823	89.3	(88.5–90.1)
Yes	763	10.2	(9.5–11.0)
**Very/completely willing to personally use political violence as part of a group of people who share your beliefs**			
No	8358	96.4	(95.9–96.9)
Yes	200	2.8	(2.4–3.2)
**Very/completely willing to personally use political violence on your own, as an individual**			
No	8199	94.4	(93.7–94.9)
Yes	364	4.9	(4.3–5.5)
**Very/completely willing to personally organize a group of people who share your beliefs to use political violence**			
No	8391	96.7	(96.2–97.1)
Yes	175	2.6	(2.2-3.0)
**Uses at least one social media platform as major source of news/information** ^c^			
No	6554	72.6	(71.5–73.7)
Yes	2008	27.4	(26.3–28.5)
**Views at least one government institution as enemy** ^d^			
No	4934	72.0	(70.9–73.1)
Yes	3603	28.0	(26.9–29.1)

Columns may not sum to total due to missing values

^a^Categories are not mutually exclusive. 62.7% of respondents self-identified as non-Hispanic White (95% CI = 61.4–65.9%), and 37.3% of respondents self-identified as non-White (Hispanic ethnicity or non-White race, including two or more races) (95% CI = 36.1–38.6%)

^b^See Supplementary Material 1: Table S1 for a list of the situations, types, and targets

^c^See Supplementary Material 1: Table S1 and the methods section for a list of social media platforms

^d^See Supplementary Material 1: Table S1 and the methods section for a list of government institutions

Most respondents considered non-political violence usually/always justified in at least one situation (86.5%, 95% CI = 85.7–87.4%, Table 1). While 3.0% of respondents considered political violence usually/always justified in general (95% CI = 2.6–3.6%), 32.8% considered political violence usually/always justified in at least one specific situation (95% CI = 31.7–33.9%). One in 10 respondents were personally willing to use violence to advance an important political objective that they support for at least one type or target of violence presented (10.2%, 95% CI = 9.5–11.0%). Less than 5% of respondents were very/completely willing to use political violence as part of a group of people who share their beliefs (2.8%, 95% CI = 2.4–3.2%), use political violence on their own as an individual (4.9%, 95% CI = 4.3–5.5%), or organize a group of people who share their beliefs to use political violence (2.6%, 95% CI = 2.2-3.0%). Descriptions of outcomes and modifiers by social network size are in Supplementary Material 1: Table S2.

### Association between social network size and violence

In adjusted models, those with 10–19 strong social connections were more likely to consider non-political violence usually/always justified in at least one situation compared with those with 1–4 strong social connections (aPR = 1.04, 95% CI = 1.01–1.06) (Table 2). Those with zero strong connections were more likely than those with 1–4 strong social connections to consider political violence usually/always justified in general (aPR = 2.43, 95% CI = 1.47–4.01). Those with 50 + strong connections were more likely than those with 1–4 strong social connections to consider political violence usually/always justified in at least one situation (aPR = 1.19, 95% CI = 1.03–1.37) and were more likely to report being willing to personally use political violence (aPR = 1.52, 95% CI = 1.13–2.04).

**Table 2:** Association between social network size and violence

	PR (95% CI)	aPR (95% CI)^a^
*Support for violence*		
**Violence**,** in 1 + situations**		
Social connections		
0	0.95 (0.89, 1.01)	0.95 (0.89, 1.02)
1–4 (Ref.)	--	--
5–9	1.02 (1.00, 1.05)	1.02 (0.99, 1.04)
10–19	1.04 (1.02, 1.07)	1.04 (1.01, 1.06)
20–49	1.02 (0.99, 1.05)	1.01 (0.97, 1.04)
50+	0.98 (0.93, 1.03)	0.96 (0.91, 1.01)
**Political violence**,** in general**		
Social connections		
0	2.96 (1.87, 4.69)	2.43 (1.47, 4.01)
1–4 (Ref.)	--	--
5–9	0.59 (0.38, 0.93)	0.73 (0.46, 1.16)
10–19	0.62 (0.36, 1.06)	0.80 (0.47, 1.37)
20–49	0.72 (0.38, 1.37)	1.08 (0.57, 2.05)
50+	1.18 (0.52, 2.65)	1.43 (0.67, 3.02)
**Political violence**,** in 1 + situations**		
Social connections		
0	1.30 (1.11, 1.52)	1.14 (0.98, 1.34)
1–4 (Ref.)	--	--
5–9	0.94 (0.86, 1.03)	1.00 (0.92, 1.09)
10–19	0.91 (0.82, 1.01)	0.99 (0.89, 1.10)
20–49	0.94 (0.82, 1.06)	1.02 (0.90, 1.16)
50+	1.22 (1.05, 1.41)	1.19 (1.03, 1.37)
*Personal willingness to engage in political violence*		
**Political violence**,** for 1 + type or target**		
Social connections		
0	1.31 (0.96, 1.79)	1.11 (0.81, 1.52)
1–4 (Ref.)	--	--
5–9	0.72 (0.59, 0.88)	0.82 (0.67, 1.00)
10–19	0.86 (0.68, 1.08)	1.02 (0.81, 1.27)
20–49	0.82 (0.62, 1.09)	1.02 (0.76, 1.36)
50+	1.43 (1.05, 1.95)	1.52 (1.13, 2.04)
**Political violence**,** organize group**		
Social connections		
0	1.76 (0.97, 3.17)	1.37 (0.74, 2.54)
1–4 (Ref.)	--	--
5–9	0.69 (0.44, 1.09)	0.85 (0.53, 1.34)
10–19	1.02 (0.63, 1.67)	1.23 (0.75, 2.00)
20–49	1.03 (0.56, 1.87)	1.41 (0.76, 2.61)
50+	1.05 (0.50, 2.20)	1.15 (0.54, 2.44)
**Political violence**,** as individual**		
Social connections		
0	1.29 (0.79, 2.09)	1.07 (0.65, 1.76)
1–4 (Ref.)	--	--
5–9	0.88 (0.65, 1.19)	0.99 (0.73, 1.33)
10–19	0.94 (0.65, 1.35)	1.08 (0.75, 1.55)
20–49	0.97 (0.65, 1.46)	1.18 (0.79, 1.77)
50+	1.41 (0.90, 2.21)	1.43 (0.91, 2.25)
**Political violence**,** as part of group**		
Social connections		
0	1.82 (1.07, 3.09)	1.47 (0.86, 2.53)
1–4 (Ref.)	--	--
5–9	0.62 (0.41, 0.95)	0.72 (0.47, 1.10)
10–19	0.73 (0.45, 1.18)	0.88 (0.54, 1.44)
20–49	0.93 (0.52, 1.67)	1.19 (0.66, 2.15)
50+	1.04 (0.54, 1.98)	1.11 (0.57, 2.14)

PR = prevalence ratio. aPR = adjusted prevalence ratio. CI = confidence interval

^a^Adjusted for age, gender, income, education, employment, and political party affiliation

### Modification of the association between social network size and violence

**Fig. 2:**
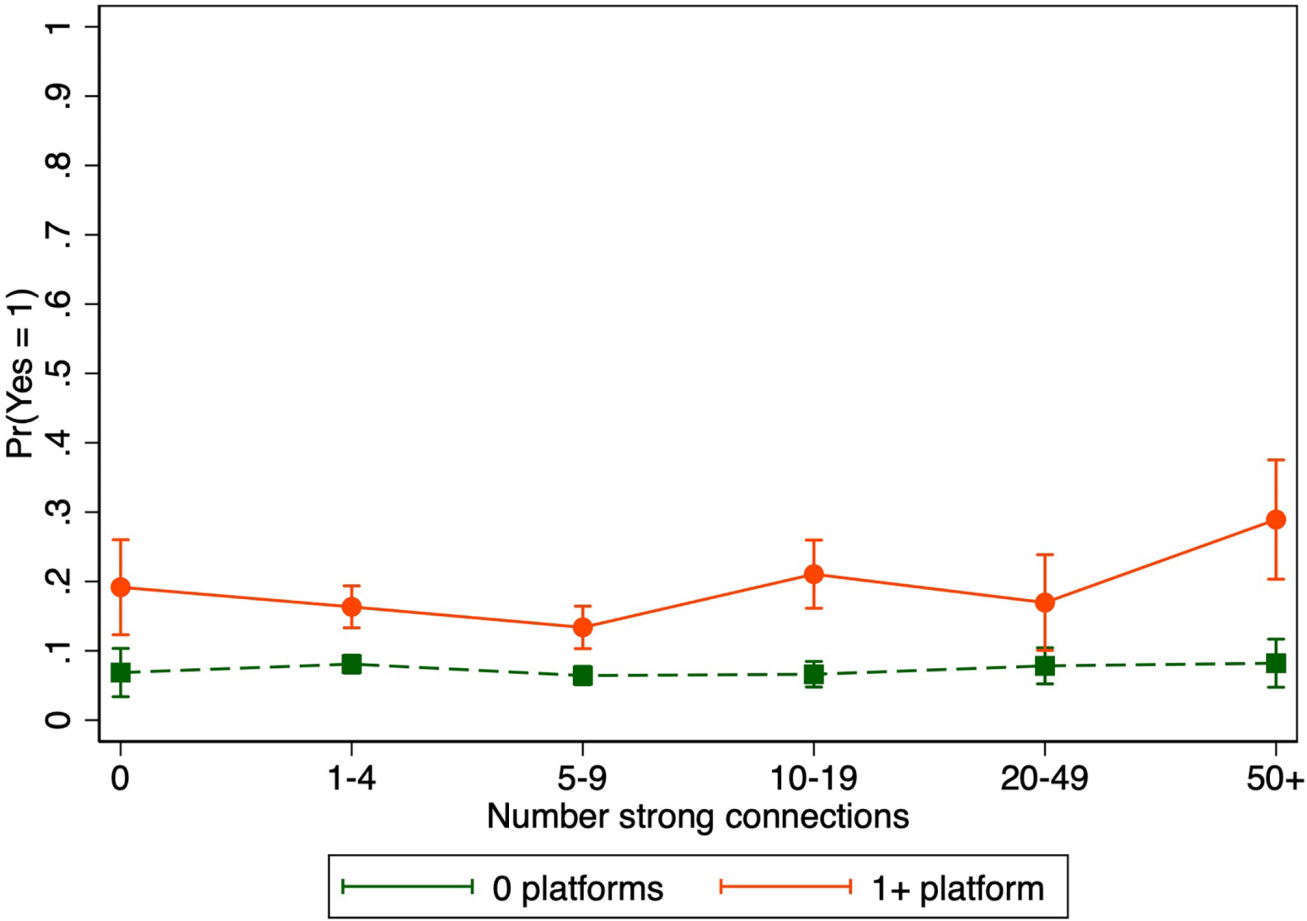
Association between social network size and personal willingness to engage in political violence by use of social media as a major source of news. Bars indicate 95% confidence intervals. Adjusted for age, gender, income, education, employment, and political party affiliation. Interaction p-value = 0.18

**Fig. 3:**
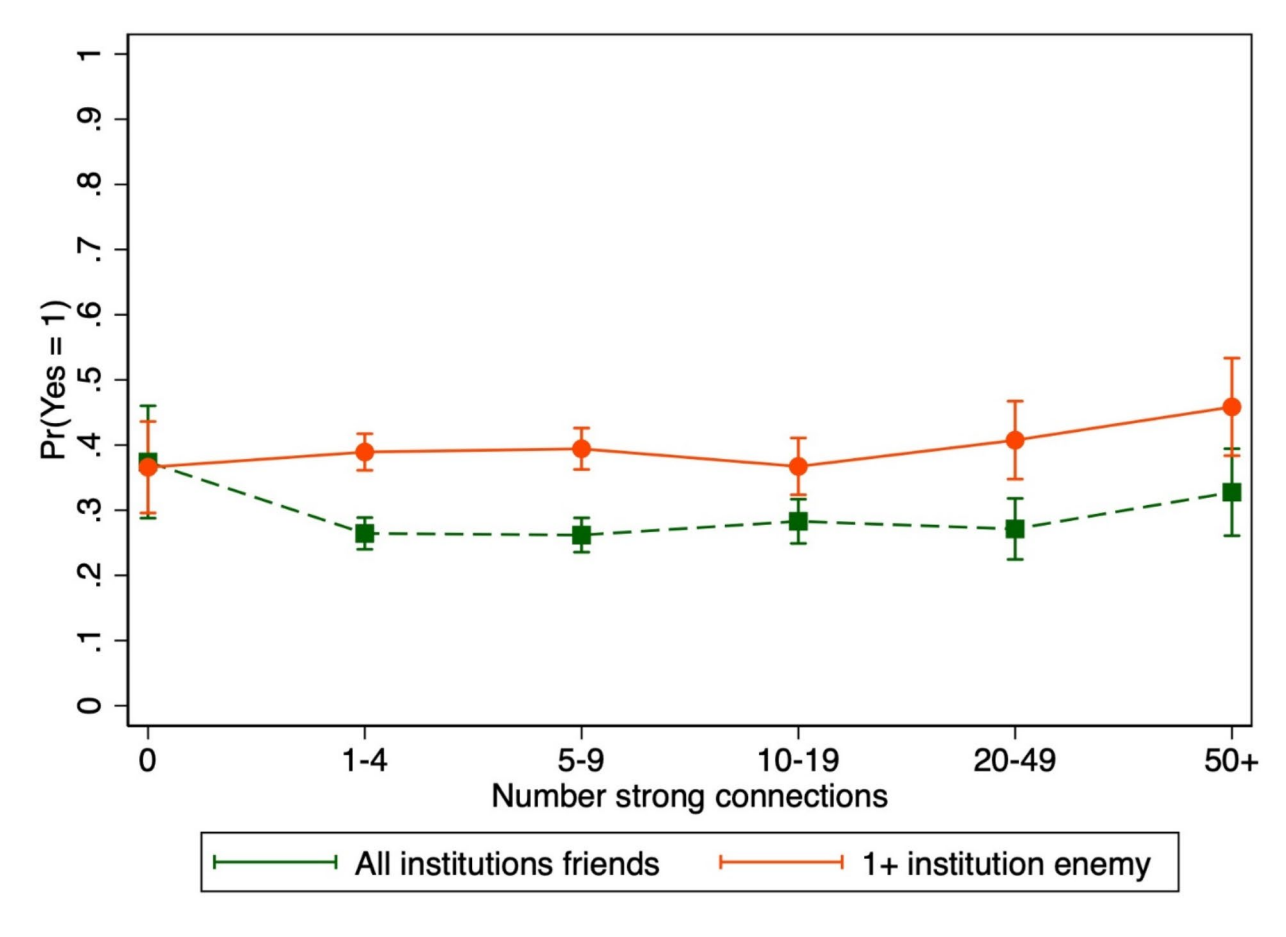
Association between social network size and endorsement of political violence as usually/always justified in at least one situation by perceptions of government institutions as an enemy. Bars indicate 95% confidence intervals. Adjusted for age, gender, income, education, employment, and political party affiliation. Interaction p-value = 0.12

**Fig. 4:**
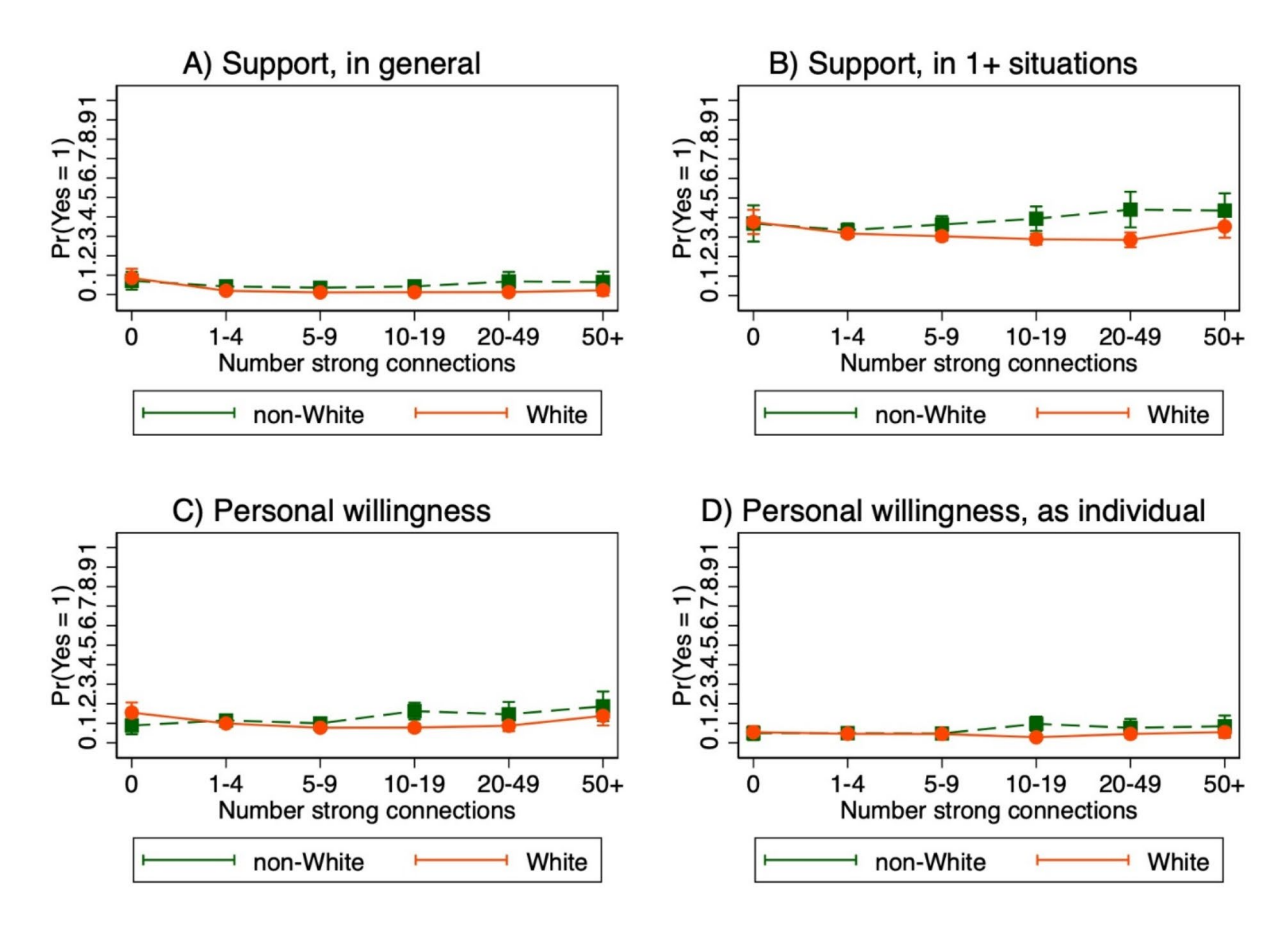
Association between social network size and political violence by membership in marginalized or privileged racial or ethnic group. Bars indicate 95% confidence intervals. Adjusted for age, gender, income, education, employment, and political party affiliation. Interaction p-values panel A = 0.09; B = 0.04, C = 0.007, D = 0.01. Non-White indicates non-White race or Hispanic ethnicity. White indicates White race and non-Hispanic ethnicity

Significant interactions at alpha < 0.20 are shown in Figs. 2, 3 and 4 and Supplementary Material 1: Table S3. There was a positive association between reporting 50+ (vs. 1–4) strong social connections and personal willingness to engage in political violence among those who reported at least one social media platform as a major source of news (aPR = 1.77, 95% CI = 1.25–2.51), but not among those who did not report any social media platform as a major source of news (Fig. 2; interaction p-value = 0.18).

There was a positive association between reporting zero (vs. 1–4) social connections and support for political violence in at least one situation among those who did not report any government institution as an enemy (aPR = 1.41, 95% CI = 1.10–1.81) but no associations among those who reported at least one government institution as an enemy (Fig. 3, Supplementary Material 1: Table S3, interaction p-value = 0.12).

There was a positive association between reporting zero (vs. 1–4) social connections and three violence outcomes—support for political violence in general (aPR = 4.31, 95% CI = 2.23–8.34, interaction p-value = 0.09), support for political violence in at least one situation (aPR = 1.19, 95% CI = 1.00-1.41, interaction p-value = 0.04), and personal willingness to engage in political violence (aPR = 1.56, 95% CI = 1.09–2.24, interaction p-value = 0.007)—among non-Hispanic White respondents (Fig. 4, Supplementary Material 1: Table S3). In contrast, there was a positive association between reporting 50+ (vs. 1–4) strong social connections and support for political violence in at least one situation (aPR = 1.30, 95% CI = 1.03–1.63, interaction p-value = 0.04) and personal willingness to engage in political violence (aPR = 1.63, 95% CI = 1.03–2.56, interaction p-value = 0.007) among non-White respondents.

### Secondary and sensitivity analyses

Despite some variability and lower power, results remained strikingly consistent when examining associations between social network size and specific situations in which respondents might justify political violence and specific types and targets of violence for which respondents might be personally willing to engage in political violence (Supplementary Material 1: Tables S4-S5). Compared with those who reported 1–4 strong connections with people with heterogenous beliefs, those who reported 5–9 and 10–19 strong social connections were more likely to endorse non-political violence, regardless of shared beliefs (Supplementary Material 1: Table S6). Those who reported 1–4 strong social connections with people who mostly share beliefs about political violence were also more likely to endorse non-political violence. There was a negative association between endorsement of political violence in at least one situation and reporting 1–4, 5–9, and 10–19 strong connections with people who mostly share beliefs about political violence. In contrast, there were positive associations between endorsement of political violence in general and reporting zero strong connections, and personal willingness to engage in political violence and reporting 50 + strong connections with people who mostly share beliefs about political violence.

Results excluding 447 individuals who indicated that any of two fake social media platforms were a source of news and information were largely consistent with the main analysis (Supplementary Material 1: Table S7).

Several differences arose when conducting effect measure modification analyses using the alternative definition of perceiving the government as an enemy, including that there was a positive association between reporting zero strong connections and willingness to engage in political violence as part of a group among those who perceived at least one government institution as an enemy (aPR = 3.51, 95% CI = 1.49–8.28, Supplementary Material 1: Table 3, interaction p-value = 0.18). Findings also varied somewhat when using continuous versions of modifiers. For example, for some outcomes, associations of large social networks and violence tended to be stronger among those who endorsed a greater numbers of social media platforms as news sources and government institutions as enemies (Supplementary Material 1: Table S3, Figs. S1–S2).

## Discussion

This nationally representative cross-sectional study found that those who reported no strong social connections (3.7% of respondents) were more likely than others to endorse political violence in general but were on average not significantly more willing to engage in it. At the other end of the spectrum, individuals who reported 50 + strong social connections (4.2% of respondents) were also more likely to endorse political violence in specific situations and to be personally willing to use political violence.

Positive associations between an absence of strong connections and support for political violence were driven primarily by non-Hispanic White respondents and those who did not report the government as an enemy. These findings may be explained by several factors. For example, socially-isolated non-Hispanic White individuals may be more likely than other non-Hispanic White individuals to experience anger and resentment at their lack of social connection and perceived threats to their privileged social position and thus lash out in the form of political violence (Bonilla-Silva 2019). Prior work suggests similar etiologic associations between social isolation and mass shootings and other forms of violence, including intimate partner violence (CDC 2024; Peterson et al. 2024). Our findings about the interaction of social network size with perceptions of government were somewhat sensitive to variable operationalization; however, the positive association between an absence of strong connections and support for political violence among those who did not perceive the government as an enemy, combined with the fact that those who saw the government as an enemy were more likely to endorse political violence across the board, suggests that perceiving the government as a friend does not protect socially-isolated individuals from supporting political violence, as it may others.

The positive associations we found between reporting 50 + strong connections and personal willingness to engage in political violence were driven by those who reported at least one social media platform as a major source of news and those who identified as non-White (the association among non-Hispanic White respondents was also large and positive but not significant). Further, a positive association between an absence of strong connections and personal willingness to engage in political violence emerged among respondents who perceived the government as an enemy (though results varied by variable operationalization) and those who identified as non-Hispanic White. These findings might reflect the ways that individual and group identity intersects with perceived grievance, desire for belongingness, and outgroup hate, and potentially evolves in larger social networks, either online or in person, to promote violence (Kleinfeld 2021; Littman and Paluck 2015).

Our findings have implications for prevention of political violence. First, they suggest that existing interventions to prevent politically motivated violent ideologies, beliefs, and behaviors should be geared towards those with very few and very many strong social connections. Our results further suggest specific opportunities for such intervention. For example, given that individuals who both reported having large social networks and using social media as a major source of news/information were more willing than others to personally engage in political violence, targeted messaging or other interventions on social media platforms can be used to reduce risk of political violence (van der Linden 2022).

Further, our results suggest that increasing prosocial connections and social cohesion—specifically among people currently experiencing social isolation and perceived marginalization or alienation—could be effective in preventing politically motivated violence and promoting productive political discourse. This aligns with prior work showing that social cohesion and collective efficacy are important protective factors for multiple forms of violence (Capaldi et al. 2012; Sampson et al. 1997), and that interventions geared toward the social and physical environment (e.g., community centers, parks, community mobilization, cultural activities, etc.) may help promote prosocial connections and sense of belonging (Breedvelt et al. 2022; Ohmer 2016).

While more research is needed to understand the roots of people’s alienation from government, addressing the crisis of loneliness, making people feel heard, protected, and socially and economically stable are potentially fruitful avenues to decrease such structural alienation (Mann et al. 2017; Stewart et al. 2021; U.S. Surgeon General 2023). Likewise, reducing racial oppression and disparities and addressing racialized resentment and anger among non-Hispanic White Americans (e.g., by acknowledging the structural roots of racism, working towards “cognitive” and “emotional liberation” from White supremacist culture, reducing racial residential and social segregation, and fostering collectivism) may help prevent political violence and promote a just, equitable, and thriving society (Bonilla-Silva 2017, 2019; Reeping et al. 2024). For example, prior studies and intergroup contact theory suggest that interventions that attempt to find elements of shared identity across groups can reduce polarization and prejudice (Kleinfeld 2024). Importantly, research indicates that interventions to bridge social differences may have limited effect on political violence if broader social and political norms and incentives remain intensely polarized and social inequities remained deeply entrenched (Kleinfeld 2024; Stewart et al. 2021).

To reduce the risk of political violence for those embedded in large social networks, interventions, administered through social media or elsewhere, that disrupt amplification of violent language, norms, and group identity (Littman and Paluck 2015) and work to shift networks towards ideas of humanization and peaceful, democratic expression of anger and calls for social change may hold promise. Certain individuals, particularly those with high status or influence, in networks that are large but heterogeneous in beliefs about political violence could also leverage their social position and act as trusted ambassadors for anti-violence (Paluck and Chwe  2017).

This study has limitations. While we asked about the degree to which individuals’ social connections shared their beliefs about political violence, this information is only ipso facto related to outcomes of political violence. Particularly for large, potentially heterogenous, social networks, additional information on the characteristics of social networks could add important nuance. We asked about specific contexts in which individuals may support or be willing to engage in political violence—responses may have changed if different contexts had been provided (Westwood et al. 2022). This survey is cross-sectional, precluding analyses of directionality and causality, and it is subject to sampling error and other biases common in survey research (e.g., social desirability). World events occurring around the time of survey administration (e.g., widely publicized mass shootings in Buffalo, NY and Uvalde, TX, Russia’s war against Ukraine) may have also affected some respondents’ views of violence and thus generalizability of our results.

## Conclusions

Understanding risk for support for and willingness to engage in political violence is a public health priority, both for political violence’s potential direct impacts of on health (e.g., injury) and its indirect impacts on health via political and social determinants. This study found that individuals who reported very few and very many strong social connections were more likely than others to support political violence or be personally willing to engage in it in one form or another. Findings point toward potential intervention and prevention opportunities.

## Electronic supplementary material

Below is the link to the electronic supplementary material.

Supplementary Material 1

## Data Availability

The datasets generated and/or analyzed during the current study are not publicly available as analyses are continuing but will be made available to qualified researchers subject to the terms of a data use agreement.
