# Supplementary material for: Social network size and endorsement of political violence in the US
Source: Inj Epidemiol. 2024 Oct 17;11:56. doi: 10.1186/s40621-024-00540-2 (PMC11488229; doi:10.1186/s40621-024-00540-2)
Supplement: Supplementary file 1 — Supplementary Material 1 [file 40621_2024_540_MOESM1_ESM.docx]

**Additional File**

Social Network Size and Endorsement of Political Violence in the US

Julia P. Schleimer^a,b^ Paul M. Reeping,^a,b^ Sonia L. Robinson,^a,b^ Garen J. Wintemute^a,b^

^a^University of California, Davis Violence Prevention Research Program, Department of Emergency Medicine, Sacramento, CA, USA

^b^California Firearm Violence Research Center, Sacramento, CA, USA

**Contents**

Supplementary Table 1. Survey Questions and Response Options

Supplementary Table 2. Description of Outcomes and Modifiers by Social Network Size

Supplementary Table 3. Association Between Social Network Size and Violence by Hypothesized Modifiers

Supplementary Table 4. Association Between Social Network Size and Support for Political Violence, by Situation

Supplementary Table 5. Association Between Social Network Size and Personal Willingness to Engaged in Political Violence, by Type and Target

Supplementary Table 6. Association Between Social Network Size and Shared Beliefs about Political Violence with Violence

Supplementary Table 7. Association Between Social Network Size and Violence, Excluding Those Who Endorsed Fake Social Media Platform, N=8,502

Supplementary Figure 1. Association Between Social Network Size and Personal Willingness to Engage in Political Violence by Use of Social Media as a Major Source of News, Continuous Operationalization

Supplementary Figure 2. Association Between Social Network Size and Political Violence by Perceptions of Government Institutions as an Enemy, Continuous Operationalization

Supplementary Table 1. Survey Questions and Response Options

| Question text | Response options | Notes |
| --- | --- | --- |
| **Exposures** |  |  |
| Q16A: This question and the next one are about people with whom you have a strong connection—the people with whom you have a personal or work relationship and communicate with regularly. How many people are there, other than yourself, with whom you have a strong connection? | 1. 0  2. 1-4  3. 5-9  4. 10-19  5. 20-49  6. 50 or more | Asked of all respondents |
| Q16B: Thinking again about the people with whom you have a strong connection, what percentage of them share your beliefs about the use of force or violence to advance important political objectives that they support—is it… | 1. None (or almost none) of them  2. Less than half of them  3. About half of them  4. More than half of them  5. All (or nearly all) of them  6. Don’t know | Asked of all respondents  Order of response options randomized (negative to positive or vice versa), with order consistent within respondent across questions |
| **Outcomes** |  |  |
| *Now we have a few questions about the use of force or violence. A reminder: your responses will be kept confidential and anonymous*.  Q11: In general, what do you think about the use of force or violence in the following situations—is it [*INSERT*: never justified, sometimes justified, usually justified, or always justified *OR INSERT*: always justified, usually justified, sometimes justified, or never justified]? “Force or violence” means physical force strong enough that it could cause pain or injury to a person.  a. In self defense  b. To prevent someone from injuring or killing another person  c. To prevent someone from injuring or killing themselves  d. To prevent harm or damage to property  e. To win an argument  f. In response to an insult  g. To get respect | 1. Never justified  2. Sometimes justified  3. Usually justified  4. Always justified | Asked of all respondents  Order of response options randomized (negative to positive or vice versa), with order consistent within respondent across questions |
| Q12: People sometimes talk about using force or violence to achieve political objectives. In general, what do you think about using force or violence to advance an important political objective that you support—is it…? | 1. Never justified  2. Sometimes justified  3. Usually justified  4. Always justified | Asked of all respondents  Order of response options randomized (negative to positive or vice versa), with order consistent within respondent across questions |
| Q13: You said that in general, the use of force or violence was **[Insert option item selected in Q12]** to advance an important political objective that you support. Your opinion might depend on the specific objective that was involved. What do you think about the use of force or violence in the following situations—is it [*INSERT*: never justified, sometimes justified, usually justified, or always justified *OR INSERT*: always justified, usually justified, sometimes justified, or never justified]?  1a. To stop voter fraud  **OR**  1b. To stop voter intimidation  2a. To stop police violence  **OR**  2b. To reinforce the police  3a. To stop illegal immigration  **OR**  3b. To keep our borders open  4a. To stop a protest or demonstration  **OR**  4b. To support a protest or demonstration  **AND**  5. To preserve the American way of life l believe in  6. To oppose Americans who do not share my beliefs | 1. Never justified  2. Sometimes justified  3. Usually justified  4. Always justified | Asked of respondents who answered question 12  Order of response options randomized (negative to positive or vice versa), with order consistent within respondent across questions  Statements randomized  Situations 1-4 were randomized, such that each respondent saw one version |
| Q13A: Again, your view of the use of force or violence to advance an important political objective might depend on the specific objective that was involved. What do you think about the use of force or violence in the following situations—is it [*INSERT*: never justified, sometimes justified, usually justified, or always justified *OR INSERT*: always justified, usually justified, sometimes justified, or never justified]?  a. To return Donald Trump to the presidency this year  b. To stop an election from being stolen  c. To stop people who do not share my beliefs from voting  d. To prevent discrimination based on race or ethnicity  e. To preserve an American way of life based on Western European traditions  f. To oppose the government when it does not share my beliefs  g. To oppose the government when it tries to take private land for public purposes | 1. Never justified  2. Sometimes justified  3. Usually justified  4. Always justified | Asked of respondents who answered question 12  Order of response options randomized (negative to positive or vice versa), with order consistent within respondent across questions    Statements randomized |
| Q13B: You didn’t answer the previous question on the use of force or violence to advance an important political objective that you support. Your opinion might depend on the specific objective that was involved. What do you think about the use of force or violence in the following situations—is it [*INSERT*: never justified, sometimes justified, usually justified, or always justified *OR INSERT*: always justified, usually justified, sometimes justified, or never justified]?  1a. To stop voter fraud  **OR**  1b. To stop voter intimidation  2a. To stop police violence  **OR**  2b. To reinforce the police  3a. To stop illegal immigration  **OR**  3b. To keep our borders open  4a. To stop a protest or demonstration  **OR**  4b. To support a protest or demonstration  **AND**  5. To preserve the American way of life l believe in  6. To oppose Americans who do not share my beliefs | 1. Never justified  2. Sometimes justified  3. Usually justified  4. Always justified | Asked of respondents who did not answer question 12  Order of response options randomized (negative to positive or vice versa), with order consistent within respondent across questions  Statements randomized  Situations 1-4 were randomized, such that each respondent saw one version |
| Q13C: Again, your view of the use of force or violence to advance an important political objective might depend on the specific objective that was involved. What do you think about the use of force or violence in the following situations—is it [INSERT: never justified, sometimes justified, usually justified, or always justified OR INSERT: always justified, usually justified, sometimes justified, or never justified]?  a. To return Donald Trump to the presidency this year  b. To stop an election from being stolen  c. To stop people who do not share my beliefs from voting  d. To prevent discrimination based on race or ethnicity  e. To preserve an American way of life based on Western European traditions  f. To oppose the government when it does not share my beliefs  g. To oppose the government when it tries to take private land for public purposes | 1. Never justified  2. Sometimes justified  3. Usually justified  4. Always justified | Asked of respondents who did not answer question 12  Order of response options randomized (negative to positive or vice versa), with order consistent within respondent across questions  Statements randomized |
| Q14: The next questions are about your personal willingness to use force or violence.  You agreed that the use of force or violence could be justified to advance [one/some] of the political objectives we just discussed. In [that/those] [situation/situations], how willing would you personally be to…  a. Use force or violence as part of a group of people who share your beliefs  b. Use force or violence on your own, as an individual  c. Organize a group of people who share your beliefs to use force or violence | 1. Not willing  2. Somewhat willing  3. Very willing  4. Completely willing | Asked of respondents who indicated that political violence was ever justified (answered somewhat, very, or completely willing to any of questions 13, 13A, 13B, or 13C); we coded those who indicated that political violence was never justified (and thus not asked this question) as unwilling to personally use political violence  Order of response options randomized (negative to positive or vice versa), with order consistent within respondent across questions  Statements randomized |
| Q15: In a situation where you think force or violence is justified to advance an important political objective, how willing would you personally be to use force or violence in each of these ways?  a. To damage property  b. To threaten or intimidate a person  c. To injure a person  d. To kill a person | 1. Not willing  2. Somewhat willing  3. Very willing  4. Completely willing | Asked of respondents who indicated that political violence was ever justified (answered somewhat, very, or completely willing to any of questions 13, 13A, 13B, or 13C); we coded those who indicated that political violence was never justified (and thus not asked this question) as unwilling to personally use political violence  Order of response options randomized (negative to positive or vice versa), with order consistent within respondent across questions |
| Q16: In a situation where you think force or violence is justified to advance an important political objective, how willing would you personally be to use force or violence against a person because they are…  a. An elected federal or state government official  b. An elected local government official  c. A public health official  d. A member of the military or National Guard  e. A police officer  f. A person who does not share your race or ethnicity  g. A person who does not share your religion  h. An election worker, such as a poll worker or vote counter  i. A person who does not share your political beliefs | 1. Not willing  2. Somewhat willing  3. Very willing  4. Completely willing | Asked of respondents who indicated that political violence was ever justified (answered somewhat, very, or completely willing to any of questions 13, 13A, 13B, or 13C); we coded those who indicated that political violence was never justified (and thus not asked this question) as unwilling to personally use political violence  Order of response options randomized (negative to positive or vice versa), with order consistent within respondent across questions  Statements randomized |
| **Modifiers** |  |  |
| *The next few questions are about your views of American society.*  Q8B: On a scale of [*INSERT* 1 to 5 *OR INSERT* 5 to 1] — where “1” means you think the institution is your [*INSERT* enemy *OR INSERT* friend] and “5” means you think the institution is your [*INSERT* friend *OR INSERT* enemy]—where on this scale would you place yourself?  a. Federal government  b. State government  c. Local government  d. Police and sheriffs  e. Courts and judges  f. Military and National Guard  g. State and local health departments | 1. 1  2. 2  3. 3  4. 4  5. 5 | Asked of all respondents  Order of response options randomized (negative to positive or vice versa), with order consistent within respondent across questions  Statements randomized |
| *The next few questions are about your sources of news and information*.  Q9: How much do you use each of the following as a source of news and information? Is it [INSERT: not a source, a minor source, or a major source OR INSERT a major source, a minor source, or not a source]?  a. Fox News  b. CNN or MSNBC  c. Broadcast TV networks, such as ABC, CBS, and NBC, or local TV news  d. Daily newspapers, such as your local paper, The New York Times, or USA Today  e. Public TV or radio, such as PBS or NPR  f. Newsmax or One America News Network  g. Conservative talk radio or podcasts, such as the Sean Hannity Show  h. Progressive talk radio or podcasts, such as Democracy Now!  i. Infowars | 1. Not a source  2. Minor source  3. Major source | Asked of all respondents  Order of response options randomized (negative to positive or vice versa), with order consistent within respondent across questions  Statements randomized |
| *Q10:* How much do you use each of the following Internet sites and apps as a source of news and information? Is it [*INSERT*: not a source, a minor source, or a major source *OR INSERT* a major source, a minor source, or not a source]?  a. Facebook/Meta  b. Twitter  c. LinkedIn  d. Parler  e. YouTube  f. Instagram  g. TikTok  h. Reddit  i. XenonExtra | 1. Not a source  2. Minor source  3. Major source | Asked of all respondents  Order of response options randomized (negative to positive or vice versa), with order consistent within respondent across questions  Statements randomized  XenonExtra was a fake internet site/app used solely for attention check |
| *Q10A:* Here are some more Internet sites and apps. How much do you use each as a source of news and information? Is it [*INSERT*: not a source, a minor source, or a major source *OR INSERT* a major source, a minor source, or not a source]?  a. Rumble  b. 8chan/8kun  c. Telegram  d. Whatsapp  e. Signal  f. Truth Social  g. TalkTrue  h. Gab | 1. Not a source  2. Minor source  3. Major source | Asked of all respondents  Order of response options randomized (negative to positive or vice versa), with order consistent within respondent across questions  Statements randomized  TalkTrue was a fake internet site/app used solely for attention check |

Supplementary Table 2. Description of Outcomes and Modifiers by Social Network Size

|  | **Number of Strong Social Connections** | | | | | | | | | | | | | | | | | |
| --- | --- | --- | --- | --- | --- | --- | --- | --- | --- | --- | --- | --- | --- | --- | --- | --- | --- | --- |
|  | **0** | | | **1-4** | | | **5-9** | | | **10-19** | | | **20-49** | | | **50+** | | |
|  | **No.^a^** | **%^b^** | **CI^b^** | **No.^a^** | **%^b^** | **CI^b^** | **No.^a^** | **%^b^** | **CI^b^** | **No.^a^** | **%^b^** | **CI^b^** | **No.^a^** | **%^b^** | **CI^b^** | **No.^a^** | **%^b^** | **CI^b^** |
| Total | 288 | 3.7 | (3.3-4.3) | 2830 | 34.5 | (33.3-35.6) | 2615 | 30 | (28.9-31.1) | 1650 | 18.3 | (17.4-19.2) | 755 | 7.9 | (7.3-8.5) | 386 | 4.2 | (3.7-4.7) |
| Consider violence usually/always justified in at least one situation | | | | | | | | | | | | | | | | | | |
| No | 50 | 18.3 | (13.8-23.9) | 349 | 13.7 | (12.3-15.3) | 286 | 11.8 | (10.4-13.3) | 174 | 10.2 | (8.7-11.9) | 90 | 12.1 | (9.8-14.9) | 57 | 15.5 | (11.9-20.1) |
| Yes | 236 | 80.7 | (75.0-85.4) | 2470 | 85.8 | (84.2-87.3) | 2321 | 87.8 | (86.3-89.3) | 1472 | 89.6 | (87.9-91.1) | 663 | 87.6 | (84.8-90.0) | 325 | 83.3 | (78.7-87.1) |
| Consider political violence usually/always justified in general | | | | | | | | | | | | | | | | | | |
| No | 256 | 88.1 | (83.1-91.7) | 2758 | 96.5 | (95.5-97.3) | 2571 | 97.8 | (96.9-98.5) | 1629 | 97.9 | (96.6-98.7) | 740 | 97.4 | (95.5-98.5) | 376 | 95.8 | (91.4-98.0) |
| Yes | 28 | 9.9 | (6.7-14.4) | 70 | 3.4 | (2.6-4.4) | 40 | 2 | (1.4-2.9) | 21 | 2.1 | (1.3-3.4) | 14 | 2.5 | (1.4-4.4) | 9 | 4 | (1.8-8.5) |
| Consider political violence usually/always justified in at least one situation^c^ | | | | | | | | | | | | | | | | | | |
| No | 157 | 56.6 | (50.1-62.9) | 1891 | 66.5 | (64.5-68.5) | 1801 | 68.5 | (66.4-70.5) | 1185 | 69.7 | (67.0-72.2) | 515 | 68.7 | (65.0-72.2) | 236 | 59.3 | (53.6-64.8) |
| Yes | 131 | 43.4 | (37.1-49.9) | 939 | 33.5 | (31.5-35.5) | 814 | 31.5 | (29.5-33.6) | 465 | 30.3 | (27.8-33.0) | 240 | 31.3 | (27.8-35.0) | 150 | 40.7 | (35.2-46.4) |
| Very/completely willing to personally use political violence for at least one type or target of violence^c^ | | | | | | | | | | | | | | | | | | |
| No | 240 | 85.1 | (80.3-88.8) | 2539 | 88.3 | (86.8-89.7) | 2422 | 91.8 | (90.4-93.0) | 1523 | 89.9 | (87.8-91.6) | 692 | 90.3 | (87.6-92.5) | 333 | 83.6 | (78.5-87.7) |
| Yes | 48 | 14.9 | (11.2-19.7) | 283 | 11.3 | (10.0-12.8) | 190 | 8.2 | (7.0-9.5) | 122 | 9.7 | (8.0-11.8) | 61 | 9.3 | (7.1-12.0) | 52 | 16.2 | (12.2-21.4) |
| Very/completely willing to personally use political violence as part of a group of people who share your beliefs | | | | | | | | | | | | | | | | | | |
| No | 267 | 93.3 | (89.7-95.7) | 2740 | 96.1 | (95.1-96.9) | 2562 | 97.8 | (97.1-98.4) | 1614 | 97.1 | (95.9-98.0) | 731 | 95.9 | (93.7-97.4) | 370 | 96.3 | (93.6-97.8) |
| Yes | 19 | 5.8 | (3.6-9.1) | 71 | 3.2 | (2.4-4.2) | 46 | 2 | (1.4-2.8) | 29 | 2.3 | (1.6-3.5) | 17 | 2.9 | (1.7-4.9) | 14 | 3.3 | (1.8-5.9) |
| Very/completely willing to personally use political violence on your own, as an individual | | | | | | | | | | | | | | | | | | |
| No | 267 | 93.1 | (89.5-95.6) | 2692 | 94.5 | (93.4-95.5) | 2507 | 95.4 | (94.3-96.3) | 1588 | 94.7 | (93.0-96.0) | 713 | 94 | (91.8-95.7) | 359 | 92.8 | (89.3-95.2) |
| Yes | 20 | 6.4 | (4.1-9.9) | 124 | 5 | (4.1-6.1) | 100 | 4.4 | (3.5-5.5) | 54 | 4.7 | (3.4-6.3) | 35 | 4.8 | (3.4-6.8) | 26 | 7 | (4.7-10.5) |
| Very/completely willing to personally organize a group of people who share your beliefs to use political violence | | | | | | | | | | | | | | | | | | |
| No | 271 | 94.5 | (91.2-96.6) | 2758 | 96.8 | (95.8-97.5) | 2565 | 98 | (97.2-98.5) | 1615 | 96.8 | (95.4-97.8) | 734 | 96.3 | (94.2-97.7) | 375 | 97 | (94.3-98.4) |
| Yes | 15 | 4.7 | (2.8-7.8) | 57 | 2.7 | (2.0-3.6) | 44 | 1.9 | (1.3-2.6) | 29 | 2.8 | (1.9-4.1) | 16 | 2.8 | (1.6-4.6) | 10 | 2.8 | (1.4-5.5) |
| Uses at least one social media platform as major source of news/information^d^ | | | | | | | | | | | | | | | | | | |
| No | 186 | 60.1 | (53.4-66.4) | 2134 | 71 | (69.0-73.0) | 2002 | 72.5 | (70.4-74.5) | 1301 | 75.2 | (72.6-77.6) | 595 | 75 | (71.2-78.5) | 281 | 68.3 | (62.6-73.5) |
| Yes | 100 | 39 | (32.7-45.7) | 687 | 28.6 | (26.7-30.7) | 607 | 27.2 | (25.2-29.3) | 343 | 24.3 | (22.0-26.9) | 159 | 24.9 | (21.4-28.7) | 101 | 30.9 | (25.7-36.6) |
| Perceives at least one government institution as an enemy^e^ | | | | | | | | | | | | | | | | | | |
| No | 131 | 45.8 | (39.4-52.3) | 1564 | 53.5 | (51.4-55.6) | 1501 | 54.8 | (52.7-57.0) | 1020 | 59 | (56.3-61.7) | 462 | 60 | (56.1-63.7) | 217 | 52.2 | (46.6-57.9) |
| Yes | 152 | 51.5 | (45.0-58.0) | 1249 | 45.8 | (43.7-47.9) | 1105 | 44.8 | (42.7-47.0) | 624 | 40.3 | (37.6-43.0) | 287 | 39.2 | (35.4-43.1) | 161 | 45.9 | (40.2-51.6) |
| Membership in marginalized racial group | | | | | | | | | | | | | | | | | | |
| Non-White | 123 | 53.4 | 47.0-59.7 | 985 | 43.3 | 41.2-45.4 | 728 | 34.8 | 32.6-37.1 | 395 | 30.5 | 27.8-33.5 | 185 | 28.1 | 24.5-32.1 | 120 | 36.2 | 30.7-42.2 |
| Non-Hispanic White | 165 | 46.6 | 40.3-53.0 | 1845 | 56.7 | 54.6-58.8 | 1887 | 65.2 | 62.9-67.4 | 1255 | 69.5 | 66.7-72.2 | 570 | 71.9 | 67.9-75.5 | 266 | 63.8 | 57.9-69.3 |
| Shared beliefs | | | | | | | | | | | | | | | | | | |
| <= Half share beliefs or DK | 273 | 94.8 | 91.1-97.0 | 1634 | 58.6 | 56.5-60.6 | 1321 | 52.4 | 50-2-54.6 | 789 | 49.9 | 47.2-52.6 | 368 | 49.6 | 45.7-53.5 | 204 | 53.4 | 47.8-59.1 |
| > Half share beliefs | 15 | 5.2 | 3.0-8.9 | 1185 | 4.1 | 39.1-43.2 | 1279 | 47.0 | 44.9-49.2 | 857 | 49.8 | 47.1-52.5 | 384 | 50.0 | 46.1-53.9 | 181 | 46.2 | 40.7-51.9 |

^a^Unweighted

^b^Weighted, column percentage

^c^See Appendix Table 1 for a list of the situations, types, and targets

^d^See Appendix Table 1 and the methods section for a list of social media platforms

^e^See Appendix table 1 and the methods section for a list of government institutions

DK = don’t know

Supplementary Table 3. Association Between Social Network Size and Violence by Hypothesized Modifiers

|  | **Violence, support in 1+ situations** | **Political violence, support in general** | **Political violence, support in 1+ situations** | **Political violence, personal willingness for 1+ type or target** | **Political violence, personal willingness to organize group** | **Political violence, personal willingness as individual** | **Political violence, personal willingness as part of group** |
| --- | --- | --- | --- | --- | --- | --- | --- |
|  | aPR (95% CI) | aPR (95% CI) | aPR (95% CI) | aPR (95% CI) | aPR (95% CI) | aPR (95% CI) | aPR (95% CI) |
| **Social media as major source of news (binary)** | | | | | | | |
|  | NS | NS | NS | p=0.18 | NS | NS | NS |
| *Among those who did not report any social media platform as a major source of news* | | | | | | | |
| 0 |  |  |  | 0.85 (0.50, 1.44) |  |  |  |
| 1-4 (Ref.) |  |  |  |  |  |  |  |
| 5-9 |  |  |  | 0.80 (0.61, 1.03) |  |  |  |
| 10-19 |  |  |  | 0.82 (0.59, 1.13) |  |  |  |
| 20-49 |  |  |  | 0.97 (0.67, 1.40) |  |  |  |
| 50+ |  |  |  | 1.02 (0.65, 1.60) |  |  |  |
| *Among those who reported at least one social media platform as a major source of news* | | | | | | | |
| 0 |  |  |  | 1.17 (0.79, 1.74) |  |  |  |
| 1-4 (Ref.) |  |  |  |  |  |  |  |
| 5-9 |  |  |  | 0.82 (0.61, 1.10) |  |  |  |
| 10-19 |  |  |  | 1.29 (0.95, 1.74) |  |  |  |
| 20-49 |  |  |  | 1.04 (0.66, 1.62) |  |  |  |
| 50+ |  |  |  | 1.77 (1.25, 2.51) |  |  |  |
| **Social media as major source of news (continuous)^a^** | | | | | | | |
|  | NS | NS | NS | p=0.05 | p=0.196 | NS | p=0.9 |
| **Perceptions of government as enemy (binary)** | | | | | | | |
|  | NS | NS | p=0.12 | NS | NS | NS | NS |
| *Among those who did not report any government institution as an enemy* | | | | | | | |
| 0 |  |  | 1.41 (1.10, 1.81) |  |  |  |  |
| 1-4 (Ref.) |  |  |  |  |  |  |  |
| 5-9 |  |  | 0.99 (0.87, 1.13) |  |  |  |  |
| 10-19 |  |  | 1.07 (0.92, 1.24) |  |  |  |  |
| 20-49 |  |  | 1.03 (0.84, 1.25) |  |  |  |  |
| 50+ |  |  | 1.24 (0.99, 1.55) |  |  |  |  |
| *Among those who reported at least one government institution as an enemy* | | | | | | | |
| 0 |  |  | 0.94 (0.77, 1.15) |  |  |  |  |
| 1-4 (Ref.) |  |  |  |  |  |  |  |
| 5-9 |  |  | 1.01 (0.91, 1.13) |  |  |  |  |
| 10-19 |  |  | 0.94 (0.82, 1.08) |  |  |  |  |
| 20-49 |  |  | 1.05 (0.89, 1.23) |  |  |  |  |
| 50+ |  |  | 1.18 (0.99, 1.41) |  |  |  |  |
| **Perceptions of government as enemy (continuous)^b^** | | | | | | | |
|  | NS | p=0.18 | p=0.004 | p=0.01 | NS | p=0.17 | p=0.05 |
| **Perceptions of government as enemy (binary), alternative definition^c^** | | | | | | | |
|  | NS | NS | NS | NS | NS | NS | p=0.18 |
| *Among those who did not report any government institution as an enemy* | | | | | | | |
| 0 |  |  |  |  |  |  | 0.92 (0.42, 2.00) |
| 1-4 (Ref.) |  |  |  |  |  |  |  |
| 5-9 |  |  |  |  |  |  | 0.56 (0.33, 0.93) |
| 10-19 |  |  |  |  |  |  | 0.73 (0.42, 1.29) |
| 20-49 |  |  |  |  |  |  | 1.11 (0.56, 2.19) |
| 50+ |  |  |  |  |  |  | 0.91 (0.37, 2.21) |
| *Among those who reported at least one government institution as an enemy* | | | | | | | |
| 0 |  |  |  |  |  |  | 3.51 (1.49, 8.28) |
| 1-4 (Ref.) |  |  |  |  |  |  |  |
| 5-9 |  |  |  |  |  |  | 1.53 (0.72, 3.25) |
| 10-19 |  |  |  |  |  |  | 1.48 (0.57, 3.87) |
| 20-49 |  |  |  |  |  |  | 1.61 (0.54, 4.75) |
| 50+ |  |  |  |  |  |  | 1.74 (0.60, 5.06) |
| **Membership in marginalized or privileged racial or ethnic group** | | | | | | | |
|  | NS | p=0.09 | p=0.04 | p=0.007 | NS | 0.01 | NS |
| *Among respondents racialized as non-Hispanic White* | | | | | | | |
| 0 |  | 4.31 (2.23, 8.34) | 1.19 (1.00, 1.41) | 1.56 (1.09, 2.24) |  | 1.17 (0.64, 2.16) |  |
| 1-4 (Ref.) |  |  |  |  |  |  |  |
| 5-9 |  | 0.58 (0.30, 1.10) | 0.96 (0.87, 1.06) | 0.78 (0.61, 0.99) |  | 0.98 (0.69, 1.37) |  |
| 10-19 |  | 0.62 (0.28, 1.40) | 0.91 (0.81, 1.02) | 0.79 (0.59, 1.04) |  | 0.61 (0.39, 0.96) |  |
| 20-49 |  | 0.66 (0.26, 1.70) | 0.90 (0.78, 1.04) | 0.88 (0.62, 1.25) |  | 0.98 (0.61, 1.60) |  |
| 50+ |  | 1.13 (0.32, 3.98) | 1.11 (0.94, 1.32) | 1.39 (0.95, 2.04) |  | 1.18 (0.67, 2.10) |  |
| *Among respondents racialized as non-White^d^* | | | | | | | |
| 0 |  | 1.68 (0.82, 3.42) | 1.10 (0.84, 1.44) | 0.77 (0.45, 1.32) |  | 1.00 (0.45, 2.20) |  |
| 1-4 (Ref.) |  |  |  |  |  |  |  |
| 5-9 |  | 0.85 (0.48, 1.51) | 1.09 (0.93, 1.27) | 0.87 (0.63, 1.20) |  | 0.97 (0.56, 1.65) |  |
| 10-19 |  | 1.00 (0.51, 1.99) | 1.17 (0.97, 1.42) | 1.41 (1.02, 1.97) |  | 1.97 (1.17, 3.30) |  |
| 20-49 |  | 1.60 (0.73, 3.53) | 1.31 (1.04, 1.66) | 1.27 (0.79, 2.04) |  | 1.55 (0.79, 3.05) |  |
| 50+ |  | 1.53 (0.63, 3.69) | 1.30 (1.03, 1.63) | 1.63 (1.03, 2.56) |  | 1.72 (0.83, 3.56) |  |

aPR = adjusted prevalence ratio. CI = confidence interval. NS = not significant at alpha <0.20.

Adjusted for age, gender, income, education, employment, and political party affiliation

^a^Results summarized graphically in Additional Figure 1.

^b^Results summarized graphically in Additional Figure 2.

^c^Defined using an alternative cutoff, with respondents categorized by whether they indicated that at least one government institution was a 1 on the friend-enemy scale vs. none (i.e., all institutions were a 2, 3, 4 or 5 on the friend-enemy scale).

^d^non-White race or Hispanic ethnicity

Supplementary Table 4. Association Between Social Network Size and Support for Political Violence, by Situation

|  | **aPR (95% CI)** |
| --- | --- |
| **To return Donald Trump to the presidency this year** |  |
| Social connections |  |
| 0 | 1.48 (1.00, 2.19) |
| 1-4 (Ref.) | -- |
| 5-9 | 1.03 (0.78, 1.35) |
| 10-19 | 0.71 (0.50, 1.02) |
| 20-49 | 1.18 (0.78, 1.79) |
| 50+ | 1.90 (1.29, 2.82) |
| **To stop an election from being stolen** |  |
| Social connections |  |
| 0 | 1.47 (1.06, 2.03) |
| 1-4 (Ref.) | -- |
| 5-9 | 0.90 (0.73, 1.11) |
| 10-19 | 0.67 (0.52, 0.88) |
| 20-49 | 1.11 (0.82, 1.49) |
| 50+ | 1.79 (1.34, 2.39) |
| **To stop people who do not share my beliefs from voting** |  |
| Social connections |  |
| 0 | 2.00 (1.17, 3.44) |
| 1-4 (Ref.) | -- |
| 5-9 | 0.90 (0.57, 1.43) |
| 10-19 | 0.70 (0.39, 1.26) |
| 20-49 | 1.30 (0.65, 2.61) |
| 50+ | 1.51 (0.73, 3.09) |
| **To prevent discrimination based on race or ethnicity** |  |
| Social connections |  |
| 0 | 0.96 (0.66, 1.40) |
| 1-4 (Ref.) | -- |
| 5-9 | 0.97 (0.80, 1.19) |
| 10-19 | 0.78 (0.61, 1.01) |
| 20-49 | 0.76 (0.55, 1.05) |
| 50+ | 1.17 (0.82, 1.69) |
| **To preserve an American way of life based on Western European traditions** |  |
| Social connections |  |
| 0 | 1.61 (1.11, 2.35) |
| 1-4 (Ref.) | -- |
| 5-9 | 0.84 (0.64, 1.10) |
| 10-19 | 0.87, 0.63, 1.22) |
| 20-49 | 0.99 (0.68, 1.46) |
| 50+ | 1.79 (1.23, 2.59) |
| **To oppose the government when it does not share my beliefs** |  |
| Social connections |  |
| 0 | 1.67 (1.04, 2.69) |
| 1-4 (Ref.) | -- |
| 5-9 | 1.03 (0.73, 1.46) |
| 10-19 | 0.52 (0.30, 0.91) |
| 20-49 | 1.06 (0.60, 1.88) |
| 50+ | 1.48 (0.86, 2.53) |
| **To oppose the government when it tries to take private land for public purposes** |  |
| Social connections |  |
| 0 | 1.29 (0.93, 1.78) |
| 1-4 (Ref.) | -- |
| 5-9 | 1.01 (0.83, 1.22) |
| 10-19 | 0.93, 0.73, 1.20) |
| 20-49 | 1.04 (0.78, 1.39) |
| 50+ | 1.57 (1.17, 2.12) |
| **To stop voter fraud^a^** |  |
| Social connections |  |
| 0 | 1.33 (0.84, 2.09) |
| 1-4 (Ref.) | -- |
| 5-9 | 1.00 (0.76, 1.32) |
| 10-19 | 0.81 (0.76, 1.32) |
| 20-49 | 1.25 (0.88, 1.80) |
| 50+ | 2.12 (1.43, 3.13) |
| **To stop voter intimidation^a^** |  |
| Social connections |  |
| 0 | 1.36 (0.90, 2.07) |
| 1-4 (Ref.) | -- |
| 5-9 | 1.16 (0.90, 1.51) |
| 10-19 | 0.93 (0.66, 1.30) |
| 20-49 | 1.12 (0.76, 1.65) |
| 50+ | 1.61 (1.08, 2.38) |
| **To stop police violence^a^** |  |
| Social connections |  |
| 0 | 1.21 (0.82, 1.79) |
| 1-4 (Ref.) | -- |
| 5-9 | 1.02 (0.81, 1.28) |
| 10-19 | 0.92 (0.69, 1.22) |
| 20-49 | 1.03 (0.71, 1.50) |
| 50+ | 1.00 (0.64, 1.57) |
| **To reinforce the police^a^** |  |
| Social connections |  |
| 0 | 1.39 (1.03, 1.89) |
| 1-4 (Ref.) | -- |
| 5-9 | 0.98 (0.83, 1.17) |
| 10-19 | 1.05 (0.85, 1.29) |
| 20-49 | 1.01 (0.78, 1.29) |
| 50+ | 1.56 (1.20, 2.03) |
| **To stop illegal immigration^a^** |  |
| Social connections |  |
| 0 | 0.95 (0.61, 1.48) |
| 1-4 (Ref.) | -- |
| 5-9 | 0.90 (0.71, 1.13) |
| 10-19 | 0.92 (0.68, 1.23) |
| 20-49 | 1.04 (0.74, 1.47) |
| 50+ | 1.50 (1.07, 2.11) |
| **To keep our borders open^a^** |  |
| Social connections |  |
| 0 | 1.33 (0.84, 2.12) |
| 1-4 (Ref.) | -- |
| 5-9 | 0.94 (0.69, 1.28) |
| 10-19 | 0.87 (0.60, 1.26) |
| 20-49 | 1.46 (0.97, 2.18) |
| 50+ | 1.53 (0.90, 2.60) |
| **To stop a protest or demonstration^a^** |  |
| Social connections |  |
| 0 | 1.34 (0.79, 2.28) |
| 1-4 (Ref.) | -- |
| 5-9 | 1.11 (0.76, 1.63) |
| 10-19 | 0.93 (0.56, 1.52) |
| 20-49 | 1.11 (0.64, 1.90) |
| 50+ | 2.28 (1.33, 3.90) |
| **To support a protest or demonstration^a^** |  |
| Social connections |  |
| 0 | 1.74 (0.96, 3.16) |
| 1-4 (Ref.) | -- |
| 5-9 | 0.86 (0.56, 1.30) |
| 10-19 | 0.63 (0.35, 1.17) |
| 20-49 | 1.89 (1.08, 3.28) |
| 50+ | 1.57 (0.80, 3.09) |
| **To preserve the American way of life I believe in** |  |
| Social connections |  |
| 0 | 1.42 (1.09, 1.86) |
| 1-4 (Ref.) | -- |
| 5-9 | 1.01 (0.86, 1.18) |
| 10-19 | 0.92 (0.76 (1.12) |
| 20-49 | 0.98 (0.77, 1.26) |
| 50+ | 1.41 (1.07, 1.86) |
| **To oppose Americans who do not share my beliefs** |  |
| Social connections |  |
| 0 | 1.42 (0.82, 2.48) |
| 1-4 (Ref.) | -- |
| 5-9 | 0.90 (0.57, 1.42) |
| 10-19 | 0.92 (0.55, 1.54) |
| 20-49 | 1.19 (0.60, 1.39) |
| 50+ | 1.64 (0.88, 3.05) |

^a^These questions were randomized, such that each respondent saw one version (see Supplementary Table 1); results are presented for the subset that saw each question.

aPR = adjusted prevalence ratio. CI = confidence interval.

Adjusted for age, gender, income, education, employment, and political party affiliation

Supplementary Table 5. Association Between Social Network Size and Personal Willingness to Engaged in Political Violence, by Type and Target

| *How willing would you personally be to use force or violence in each of these ways?* | **aPR (95% CI)** |
| --- | --- |
| **To damage property** |  |
| Social connections |  |
| 0 | 1.33 (0.73, 2.43) |
| 1-4 (Ref.) | -- |
| 5-9 | 0.71 (0.47, 1.07) |
| 10-19 | 1.04 (0.65, 1.67) |
| 20-49 | 1.41 (0.83, 2.39) |
| 50+ | 2.14 (1.21, 3.80) |
| **To threaten or intimidate a person** |  |
| Social connections |  |
| 0 | 1.88 (0.96, 3.65) |
| 1-4 (Ref.) | -- |
| 5-9 | 0.75 (0.45, 1.27) |
| 10-19 | 1.04 (0.59, 1.84) |
| 20-49 | 1.20 (0.57, 2.52) |
| 50+ | 2.32 (1.18, 4.57) |
| **To injure a person** |  |
| Social connections |  |
| 0 | 1.94 (1.03, 3.66) |
| 1-4 (Ref.) | -- |
| 5-9 | 0.94 (0.55, 1.61) |
| 10-19 | 1.32 (0.75, 2.31) |
| 20-49 | 1.08 (0.49, 2.37) |
| 50+ | 3.16 (1.67, 5.99) |
| **To kill a person** |  |
| Social connections |  |
| 0 | 1.73 (0.93, 3.25) |
| 1-4 (Ref.) | -- |
| 5-9 | 0.90 (0.57, 1.43) |
| 10-19 | 0.82 (0.45, 1.46) |
| 20-49 | 1.06 (0.53, 2.13) |
| 50+ | 2.19 (1.13, 4.24) |
| *How willing would you personally be to use force or violence against a person because they are…* |  |
| **An elected federal or state government official** |  |
| Social connections |  |
| 0 | 2.16 (1.09, 4.28) |
| 1-4 (Ref.) | -- |
| 5-9 | 0.83 (0.47, 1.45) |
| 10-19 | 1.54 (0.86, 2.74) |
| 20-49 | 1.94 (0.94 (3.99) |
| 50+ | 2.90 (1.40, 5.99) |
| **An elected local government official** |  |
| Social connections |  |
| 0 | 1.85 (0.89, 3.82) |
| 1-4 (Ref.) | -- |
| 5-9 | 0.96 (0.55, 1.67) |
| 10-19 | 0.86 (0.45, 1.62) |
| 20-49 | 0.96 (0.44, 2.10) |
| 50+ | 2.26 (1.08, 4.72) |
| **A public health official** |  |
| Social connections |  |
| 0 | 3.16 (1.67, 5.98) |
| 1-4 (Ref.) | -- |
| 5-9 | 1.03 (0.56, 1.89) |
| 10-19 | 0.74 (0.36, 1.55) |
| 20-49 | 1.42 (0.61, 3.33) |
| 50+ | 2.06 (0.91, 4.66) |
| **A member of the military or National Guard** |  |
| Social connections |  |
| 0 | 1.49 (0.78, 2.85) |
| 1-4 (Ref.) | -- |
| 5-9 | 0.68 (0.39, 1.18) |
| 10-19 | 1.02 (0.55, 1.84) |
| 20-49 | 0.63 (0.25, 1.61) |
| 50+ | 2.46 (1.20, 5.05) |
| **A police officer** |  |
| Social connections |  |
| 0 | 1.67 (0.90, 3.12) |
| 1-4 (Ref.) | -- |
| 5-9 | 0.79 (0.48, 1.30) |
| 10-19 | 1.14 (0.69, 1.88) |
| 20-49 | 1.26 (0.64, 2.46) |
| 50+ | 2.10 (0.99, 4.42) |
| **A person who does not share your race or ethnicity** |  |
| Social connections |  |
| 0 | 2.66 (1.38, 5.13) |
| 1-4 (Ref.) | -- |
| 5-9 | 0.81 (0.44, 1.51) |
| 10-19 | 1.07 (0.53, 2.16) |
| 20-49 | 1.22 (0.51, 2.93) |
| 50+ | 1.64 (0.64, 4.21) |
| **A person who does not share your religion** |  |
| Social connections |  |
| 0 | 2.08 (0.96, 4.51) |
| 1-4 (Ref.) | -- |
| 5-9 | 1.02 (0.54, 1.91) |
| 10-19 | 1.84 (0.98, 3.44) |
| 20-49 | 1.39 (0.55, 3.51) |
| 50+ | 2.29 (0.88, 5.96) |
| **An election worker, such as a poll worker or vote counter** |  |
| Social connections |  |
| 0 | 1.67 (0.82, 3.42) |
| 1-4 (Ref.) | -- |
| 5-9 | 0.65 (0.35, 1.20) |
| 10-19 | 1.02 (0.54, 1.92) |
| 20-49 | 1.03 (0.42, 2.52) |
| 50+ | 1.32 (0.53, 3.25) |
| **A person who does not share your political beliefs** |  |
| Social connections |  |
| 0 | 2.75 (1.36, 5.53) |
| 1-4 (Ref.) | -- |
| 5-9 | 0.83 (0.47, 1.56) |
| 10-19 | 1.36 (0.70, 2.64) |
| 20-49 | 1.50 (0.63, 3.55) |
| 50+ | 0.74 (0.21, 2.53) |

aPR = adjusted prevalence ratio. CI = confidence interval.

Adjusted for age, gender, income, education, employment, and political party affiliation

Supplementary Table 6. Association Between Social Network Size and Shared Beliefs about Political Violence with Violence

|  | **aPR (95% CI)** |
| --- | --- |
| *Support for violence* |  |
| **Violence, in 1+ situations** |  |
| Social connections x shared beliefs |  |
| 0 connections | 0.97 (0.91, 1.04) |
| 1-4 connections, <= half share beliefs or DK (Ref.) | -- |
| 1-4 connections, > half share beliefs (Ref.) | 1.05 (1.02, 1.09) |
| 5-9 connections, <= half share beliefs or DK | 1.04 (1.01, 1.07) |
| 5-9 connections, > half share beliefs | 1.04 (1.01, 1.08) |
| 10-19, <= half share beliefs or DK | 1.06 (1.02, 1.10) |
| 10-19 connections, > half share beliefs | 1.06 (1.02, 1.10) |
| 20-49, <= half share beliefs or DK | 1.03 (0.98, 1.08) |
| 20-49 connections, > half share beliefs | 1.03 (0.98, 1.08) |
| 50+, <= half share beliefs or DK | 0.95 (0.87, 1.02) |
| 50+ connections, > half share beliefs | 1.02 (0.96, 1.09) |
| **Political violence, in general** |  |
| Social connections x shared beliefs |  |
| 0 connections | 2.37 (1.39, 4.05) |
| 1-4 connections, <= half share beliefs or DK (Ref.) | -- |
| 1-4 connections, > half share beliefs (Ref.) | 0.93 (0.54, 1.61) |
| 5-9 connections, <= half share beliefs or DK | 0.82 (0.47, 1.45) |
| 5-9 connections, > half share beliefs | 0.57 (0.29, 1.12) |
| 10-19, <= half share beliefs or DK | 0.86 (0.46, 1.62) |
| 10-19 connections, > half share beliefs | 0.64 (0.26, 1.62) |
| 20-49, <= half share beliefs or DK | 0.76 (0.30, 1.92) |
| 20-49 connections, > half share beliefs | 1.36 (0.59, 3.13) |
| 50+, <= half share beliefs or DK | 1.54 (0.66, 3.60) |
| 50+ connections, > half share beliefs | 1.20 (0.33, 4.39) |
| **Political violence, in 1+ situations** |  |
| Social connections x shared beliefs |  |
| 0 connections | 1.10 (0.93, 1.29) |
| 1-4 connections, <= half share beliefs or DK (Ref.) | -- |
| 1-4 connections, > half share beliefs (Ref.) | 0.87 (0.78, 0.99) |
| 5-9 connections, <= half share beliefs or DK | 1.03 (0.93, 1.15) |
| 5-9 connections, > half share beliefs | 0.85 (0.75, 0.96) |
| 10-19, <= half share beliefs or DK | 1.03 (0.90, 1.17) |
| 10-19 connections, > half share beliefs | 0.84 (0.72, 0.97) |
| 20-49, <= half share beliefs or DK | 1.08 (0.92, 1.27) |
| 20-49 connections, > half share beliefs | 0.84 (0.69, 1.02) |
| 50+, <= half share beliefs or DK | 1.14 (0.94, 1.38) |
| 50+ connections, > half share beliefs | 1.11 (0.91, 1.36) |
| *Personal willingness to engage in political violence* |  |
| **Political violence, for 1+ type or target** |  |
| Social connections x shared beliefs |  |
| 0 connections | 1.07 (0.77, 1.49) |
| 1-4 connections, <= half share beliefs or DK (Ref.) | -- |
| 1-4 connections, > half share beliefs (Ref.) | 0.90 (0.69, 1.17) |
| 5-9 connections, <= half share beliefs or DK | 0.79 (0.61, 1.03) |
| 5-9 connections, > half share beliefs | 0.78 (0.59, 1.03) |
| 10-19, <= half share beliefs or DK | 1.14 (0.88, 1.49) |
| 10-19 connections, > half share beliefs | 0.76 (0.53, 1.10) |
| 20-49, <= half share beliefs or DK | 1.13 (0.78, 1.63) |
| 20-49 connections, > half share beliefs | 0.82 (0.52, 1.28) |
| 50+, <= half share beliefs or DK | 1.33 (0.89, 1.99) |
| 50+ connections, > half share beliefs | 1.61 (1.06, 2.43) |
| **Political violence, organize group** |  |
| Social connections x shared beliefs |  |
| 0 connections | 1.39 (0.72, 2.69) |
| 1-4 connections, <= half share beliefs or DK (Ref.) | -- |
| 1-4 connections, > half share beliefs (Ref.) | 1.05 (0.57, 1.93) |
| 5-9 connections, <= half share beliefs or DK | 0.95 (0.53, 1.68) |
| 5-9 connections, > half share beliefs | 0.76 (0.39, 1.47) |
| 10-19, <= half share beliefs or DK | 1.25 (0.68, 2.29) |
| 10-19 connections, > half share beliefs | 1.25 (0.60, 2.62) |
| 20-49, <= half share beliefs or DK | 1.71 (0.81, 3.60) |
| 20-49 connections, > half share beliefs | 1.12 (0.45, 3.09) |
| 50+, <= half share beliefs or DK | 0.60 (0.17, 2.12) |
| 50+ connections, > half share beliefs | 1.88 (0.71, 4.97) |
| **Political violence, as individual** |  |
| Social connections x shared beliefs |  |
| 0 connections | 1.11 (0.66, 1.87) |
| 1-4 connections, <= half share beliefs or DK (Ref.) | -- |
| 1-4 connections, > half share beliefs (Ref.) | 1.12 (0.75, 1.92) |
| 5-9 connections, <= half share beliefs or DK | 0.91 (0.61, 1.36) |
| 5-9 connections, > half share beliefs | 1.20 (0.81, 1.78) |
| 10-19, <= half share beliefs or DK | 1.22 (0.79, 1.87) |
| 10-19 connections, > half share beliefs | 1.03 (0.58, 1.81) |
| 20-49, <= half share beliefs or DK | 1.28 (0.77, 2.12) |
| 20-49 connections, > half share beliefs | 1.21 (0.66, 2.19) |
| 50+, <= half share beliefs or DK | 1.27 (0.66, 2.45) |
| 50+ connections, > half share beliefs | 1.78 (0.97, 3.30) |
| **Political violence, as part of group** |  |
| Social connections x shared beliefs |  |
| 0 connections | 1.55 (0.86, 2.79) |
| 1-4 connections, <= half share beliefs or DK (Ref.) | -- |
| 1-4 connections, > half share beliefs (Ref.) | 1.14 (0.66, 1.97) |
| 5-9 connections, <= half share beliefs or DK | 0.74, 0.42, 1.29) |
| 5-9 connections, > half share beliefs | 0.78 (0.43, 1.40) |
| 10-19, <= half share beliefs or DK | 1.11 (0.61, 2.01) |
| 10-19 connections, > half share beliefs | 0.68 (0.31, 1.50) |
| 20-49, <= half share beliefs or DK | 1.51 (0.74, 3.09) |
| 20-49 connections, > half share beliefs | 0.96 (0.36, 2.58) |
| 50+, <= half share beliefs or DK | 0.80 (0.31, 2.12) |
| 50+ connections, > half share beliefs | 1.60 (0.65, 3.94) |

aPR = adjusted prevalence ratio. CI = confidence interval. DK = don’t know.

Adjusted for age, gender, income, education, employment, and political party affiliation

Supplementary Table 7. Association Between Social Network Size and Violence, Excluding Those Who Endorsed Fake Social Media Platform, N=8,502

|  | **aPR (95% CI)** |
| --- | --- |
| *Support for violence* |  |
| **Violence, in 1+ situations** |  |
| Social connections |  |
| 0 | 0.95 (0.89, 1.02) |
| 1-4 (Ref.) | -- |
| 5-9 | 1.02 (0.99, 1.04) |
| 10-19 | 1.03 (1.01, 1.06) |
| 20-49 | 1.00 (0.97, 1.03) |
| 50+ | 0.95 (0.90, 1.00) |
| **Political violence, in general** |  |
| Social connections |  |
| 0 | 2.84 (1.51, 5.33) |
| 1-4 (Ref.) | -- |
| 5-9 | 0.67 (0.35, 1.27) |
| 10-19 | 0.75 (0.37, 1.51) |
| 20-49 | 0.76 (0.28, 2.06) |
| 50+ | 1.42 (0.46, 4.39) |
| **Political violence, in 1+ situations** |  |
| Social connections |  |
| 0 | 1.17 (0.99, 1.40) |
| 1-4 (Ref.) | -- |
| 5-9 | 1.00 (0.92, 1.10) |
| 10-19 | 0.98 (0.88, 1.09) |
| 20-49 | 0.95 (0.83, 1.09) |
| 50+ | 1.13 (0.97, 1.32) |
| *Personal willingness to engage in political violence* |  |
| **Political violence, for 1+ type or target** |  |
| Social connections |  |
| 0 | 1.22 (0.56, 1.76) |
| 1-4 (Ref.) | -- |
| 5-9 | 0.80 (0.64, 1.00) |
| 10-19 | 0.97 (0.75, 1.25) |
| 20-49 | 0.90 (0.65, 1.26) |
| 50+ | 1.39 (0.98, 1.97) |
| **Political violence, organize group** |  |
| Social connections |  |
| 0 | 1.67 (0.76, 3.64) |
| 1-4 (Ref.) | -- |
| 5-9 | 0.88 (0.49, 1.57) |
| 10-19 | 1.58 (0.87, 2.89) |
| 20-49 | 0.84 (0.35, 2.00) |
| 50+ | 1.03 (0.40, 2.63) |
| **Political violence, as individual** |  |
| Social connections |  |
| 0 | 1.15 (0.64, 2.06) |
| 1-4 (Ref.) | -- |
| 5-9 | 0.98 (0.71, 1.36) |
| 10-19 | 1.06 (0.70, 1.60) |
| 20-49 | 1.14 (0.73, 1.77) |
| 50+ | 1.30 (0.78, 2.15) |
| **Political violence, as part of group** |  |
| Social connections |  |
| 0 | 1.96 (0.96, 3.97) |
| 1-4 (Ref.) | -- |
| 5-9 | 0.99 (0.58, 1.68) |
| 10-19 | 1.18 (0.65, 2.58) |
| 20-49 | 1.03 (0.47, 2.24) |
| 50+ | 1.28 (0.61, 2.65) |

aPR = adjusted prevalence ratio. CI = confidence interval.

Adjusted for age, gender, income, education, employment, and political party affiliation

Supplementary Figure 1. Association Between Social Network Size and Personal Willingness to Engage in Political Violence by Use of Social Media as a Major Source of News, Continuous Operationalization^a^

Bars indicate 95% confidence intervals. Adjusted for age, gender, income, education, employment, and political party affiliation. Interaction p-values panel A = 0.05; B = 0.196, C = 0.09.

^a^Number of social media platforms as a major source of news ranged from 0 to 15, but we top coded this variable at 4+ as very few people reported more than 4 sources as a major source of news

Supplementary Figure 2. Association Between Social Network Size and Political Violence by Perceptions of Government Institutions as an Enemy, Continuous Operationalization^a^

Bars indicate 95% confidence intervals. Adjusted for age, gender, income, education, employment, and political party affiliation. Interaction p-values panel A = 0.18; B = 0.004, C = 0.01, D = 0.17, E=0.05.

^a^Number of government institutions viewed as an enemy ranged from 0 to 7
